# A tree-based method for the rapid screening of chemical fingerprints

**DOI:** 10.1186/1748-7188-5-9

**Published:** 2010-01-04

**Authors:** Thomas G Kristensen, Jesper Nielsen, Christian NS Pedersen

**Affiliations:** 1 Bioinformatics Research Center (BiRC), Aarhus University, CF Møllers Allé 8, DK-8000 Århus C, Denmark

## Abstract

**Background:**

The fingerprint of a molecule is a bitstring based on its structure, constructed such that structurally similar molecules will have similar fingerprints. Molecular fingerprints can be used in an initial phase of drug development for identifying novel drug candidates by screening large databases for molecules with fingerprints similar to a query fingerprint.

**Results:**

In this paper, we present a method which efficiently finds all fingerprints in a database with Tanimoto coefficient to the query fingerprint above a user defined threshold. The method is based on two novel data structures for rapid screening of large databases: the *k*D grid and the Multibit tree. The *k*D grid is based on splitting the fingerprints into *k *shorter bitstrings and utilising these to compute bounds on the similarity of the complete bitstrings. The Multibit tree uses hierarchical clustering and similarity within each cluster to compute similar bounds. We have implemented our method and tested it on a large real-world data set. Our experiments show that our method yields approximately a three-fold speed-up over previous methods.

**Conclusions:**

Using the novel *k*D grid and Multibit tree significantly reduce the time needed for searching databases of fingerprints. This will allow researchers to (1) perform more searches than previously possible and (2) to easily search large databases.

## 1 Introduction

When developing novel drugs, researchers are faced with the task of selecting a subset of all commercially available molecules for further experiments. There are more than 8 million such molecules available [1], and it is not feasible to perform computationally expensive calculations on each one. Therefore, the need arises for fast screening methods for identifying the molecules that are most likely to have an effect on a given disease. It is often the case that a molecule with some effect is already known, e.g. from an already existing drug. An obvious initial screening method presents itself, namely to identify the molecules which are similar to this known molecule. To implement this screening method one must decide on a representation of the molecules and a similarity measure between representations of molecules. Several representations and similarity measures have been proposed [2-4]. We focus on *molecular fingerprints*. A fingerprint for a given molecule is a bitstring of size *N *which summarises structural information about the molecule [3]. Fingerprints should be constructed such that if two fingerprints are very similar, so are the molecules which they represent. There are several ways of measuring the similarity between fingerprints [4]. We focus on the *Tanimoto coefficient*, which is a normalised measure of how many bits two fingerprints share. It is 1.0 when the fingerprints are the same, and strictly smaller than 1.0 when they are not. Molecular fingerprints in combination with the Tanimoto coefficient have been used successfully in previous studies [5].

We focus on the screening problem of finding all fingerprints in a database with Tanimoto coefficient to a query fingerprint above a given threshold, e.g. 0.9. Previous attempts have been made to improve the query time. One approach is to reduce the number of fingerprints in the database for which the Tanimoto coefficient to the query fingerprint has to be computed explicitly. This includes storing the fingerprints in the database in a vector of bins [6], or in a trie like structure [7], such that searching certain bins, or parts of the trie, can be avoided based on an upper-bound on the Tanimoto coefficient between the query fingerprint and all fingerprints in individual bins or subtries. Another approach is to store an XOR summary, i.e. a shorter bitstring, of each fingerprint in the database, and use these as rough upper bounds on the maximal Tanimoto coefficients achievable, before calculating the exact coefficients [8].

In this paper, we present an efficient method for the screening problem, which is based on an extension of an upper bound given in [6] and two novel tree based data structures for storing and retrieving fingerprints. To further reduce the query time we also utilise the XOR summary strategy [8]. We have implemented our method and tested it on a realistic data set. Our experiments clearly demonstrate that it is superior to previous strategies, as it yields a three-fold speed-up over the previous best method.

## 2 Methods

**Figure 1:**
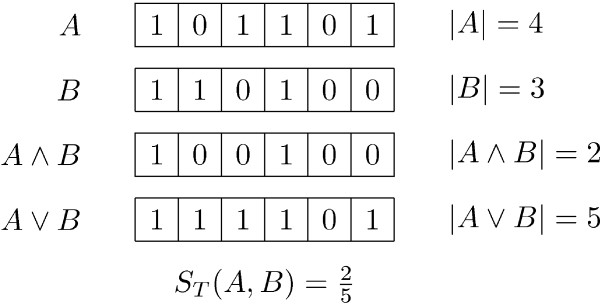
**Example calculation of Tanimoto coefficient**. Example of calculation of the Tanimoto coefficient *S*_*T*_(*A*, *B*), where *A *= 101101 and *B *= 110100.

A fingerprint is a bitstring of length *N*. Let *A *and *B *be bitstrings, and let |*A*| denote the number of 1-bits in *A*. Let *A *∧ *B *denote the *logical and *of *A *and *B*, that is, *A *∧ *B *is the bitstring that has 1-bits in exactly those positions where both *A *and *B *do. Likewise, let *A *∨ *B *denote the *logical or *of *A *and *B*, that is, *A *∨ *B *is the bitstring that has 1-bits in exactly those positions where either *A *or *B *do. With this notation the Tanimoto coefficient becomes:

Figure 1 shows an example the usage of this notation. In the following, we present a method for finding all fingerprints *B *in a database of fingerprints with a Tanimoto coefficient above some query-specific threshold *S*_min _to a query fingerprint *A*. The method is based on two novel data structures, the *k*D grid and the Multibit tree, for storing the database of fingerprints.

### 2.1 *k*D grid

Swamidass *et al*. showed in [6] that if |*A*| and |*B*| are known, *S*_*T *_(*A*, *B*) can be upper-bounded by

This bound can be used to speed up the search, by storing the database of fingerprints in *N *+ 1 buckets such that bitstring *B *is stored in the |*B*|th bucket. When searching for bitstrings similar to a query bitstring *A *it is sufficient to examine the buckets where *S*_max _≥ *S*_min_.

We have generalised this strategy. Select a number of dimensions *k *and split the bitstrings into *k *equally sized fragments such that

where *X*·*Y *is the concatenation of bitstrings *X *and *Y *.

**Figure 2:**
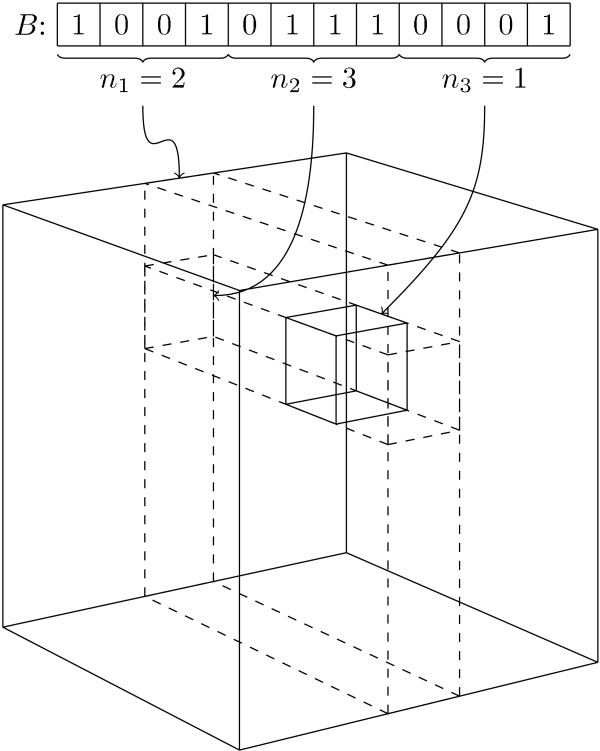
**3D grid**. Example of a *k*D-grid with *k *= 3. *B *is split into smaller substrings and the count of 1-bits in each determines where in *B *is placed in the grid. The small inner cube shows the placement of *B*.

The values |*A*_1_|, |*A*_2_|, ..., |*A*_*k*_| and |*B*_1_|, |*B*_2_|, ..., |*B*_*k*_| can be used to obtain a tighter bound than *S*_max_. Let *N*_*i *_be the length of *A*_*i *_and *B*_*i*_. The *k*D grid is a *k*-dimensional cube of size (*N*_1 _+ 1) × (*N*_2 _+ 1) × ... × (*N*_*k *_+ 1). Each grid point is a bucket and the fingerprint *B *is stored in the bucket at coordinates (*n*_1_, *n*_2_, ..., *n*_*k*_), where *n*_*i *_= |*B*_*i*_|. An example of such a grid is illustrated in Fig. 2. By comparing the partial coordinates (*n*_1_, *n*_2_, ..., *n*_*i*_) of a given bucket to |*A*_1_|, |*A*_2_|, ..., |*A*_*i*_|, where *i *≤ *k*, it is possible to upper-bound the Tanimoto coefficient between *A *and every *B *in that bucket. By looking at the partial coordinates (*n*_1_, *n*_2_, ..., *n*_*i*-1_), we can use this to quickly identify those partial coordinates (*n*_1_, *n*_2_, ..., *n*_*i*_) that may contain fingerprints *B *with a Tanimoto coefficient above *S*_min_.

Assume the algorithm is visiting a partial coordinate at level *i *in the data structure. The indices *n*_1_, *n*_2_, ..., *n*_*i*-1 _are known, but we need to compute which *n*_*i *_to visit at this level. The entries to be visited further down the data structure *n*_*i*+1_, ..., *n*_*k *_are, of course, unknown at this point. A bound can be calculated in the following manner.

The *n*_*i*_s to visit lie in an interval and it is thus sufficient to compute the upper and lower indices of this interval, *n*_*u *_and *n*_*l *_respectively. Setting , isolating *n*_*i *_and ensuring that the result is an integer in the range 0... *N*_*i *_gives:

and

where  is a bound on the number of 1-bits in the *logical and *in the first part of the bitstrings.  is a bound for the *logical or *in the first part of the bitstrings.

Similarly,  is a bound on the last part.

**Figure 3:**
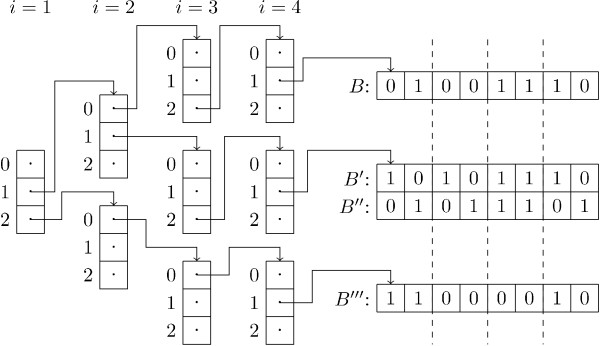
**4D grid**. Example of a 4D grid containing four bitstrings, stored as in our implementation. The dotted lines indicate the splits between *B*_*i *_and *B*_*i*+1_.

Note that in the case where *k *= 1 this datastructure simply becomes the list presented by Swamidass *et al*. [6], and in the case where *k *= *N *the datastructure becomes the binary trie presented by Smellie [7]. We have implemented the *k*D grid as a list of lists, where any list containing no fingerprints is omitted. See Fig. 3 for an example of a 4D grid containing four bitstrings. The fingerprints stored in a single bucket in the *k*D grid can be organised in a number of ways. The most naive approach is to store them in a simple list which has to be searched linearly. We propose to store them in tree structures as explained below.

### 2.2 Singlebit tree

The *Singlebit tree *is a binary tree which stores the fingerprints of a single bucket from a *k*D grid. At each node in the tree a position in the bitstring is chosen. All fingerprints with a zero at that position are stored in the left subtree while all those with a one are stored in the right subtree. This division is continued recursively until all the fingerprints in a given node are the same. When searching for a query bitstring *A *in the tree it now becomes possible, by comparing *A *to the path from the root of the tree to a given node, to compute an upper bound  on *S*_*T *_(*A*, *B*) for every fingerprint *B *in the subtree of that given node. Given two bitstring *A *and *B *let *M*_*ij *_be the number of positions where *A *has an *i *and *B *has a *j*. There are four possible combinations of *i *and *j*, namely *M*_00_, *M*_01_, *M*_10 _and *M*_11_.

The path from the root of a tree to a node defines lower limits *m*_*ij *_on *M*_*ij *_for every fingerprint in the subtree of that node. Let *u*_*ij *_denote the unknown difference between *M*_*ij *_and *m*_*ij*_, that is *u*_*ij *_= *M*_*ij*_- *m*_*ij *_.

Remember that  is known when processing a given bucket.

By using

an upper bound on the Tanimoto coefficient of any fingerprint *B *in the subtree can then be calculated as

**Figure 4:**
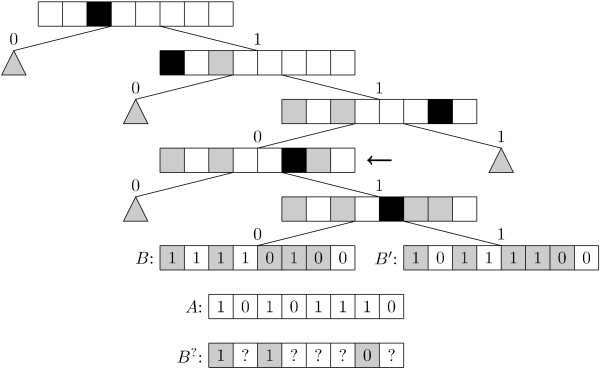
**Singlebit tree**. Example of a Singlebit tree. The black squares mark the bits chosen for the given node, while the grey squares mark bits chosen at an ancestor. The grey triangles represent subtrees omitted to keep this example simple. Assume we are searching for the bitstring *A *in the example. When examining the node marked by the arrow we have the knowledge shown in *B*^? ^about all children of that node. Comparing *A *against *B*^? ^gives us *m*_00 _= 0, *m*_01 _= 0, *m*_10 _= 1 and *m*_11 _= 2. Thus . Indeed we find that *S*_*T *_(*A*, *B*) =  and *S*_*T *_(*A, B'*) = .

When building the tree data structure it is not immediately obvious how best to choose which bit positions to split the data on, at a given node. The implemented approach is to go through all the children of the node and choose the bit which best splits them into two parts of equal size, in the hope that this creates a well-balanced tree. It should be noted that the tree structure that gives the best search time is not necessarily a well-balanced tree. Figure 4 shows an example of a Singlebit tree.

The Singlebit tree can also be used to store all the fingerprints in the database without a *k*D grid. In this case, however, |*B*| is no longer available and thus the  bound cannot be used. A less tight bound can be formulated, but experiments, not included in this paper, indicate that this is a poor strategy.

### 2.3 Multibit tree

The experiments in Sec. 3 unfortunately show that using the *k*D grid combined with Singlebit trees decreases performance compared to using the *k*D grid and simple lists. The fingerprints used in our experiments have a length of 1024 bits. In our experiments no Singlebit tree was observed to contain more the 40,000 fingerprints. This implies that the expected height of the Singlebit trees is no more than 15 (as we aim for balanced trees cf. above). Consequently, the algorithm will only obtain information about 15 out of 1024 bits before reaching the fingerprints. A strategy for obtaining more information is to store a list of bit positions, along with an annotation of whether each bit is zero or one, in each node. The bits in this list are called the *match-bits*.

The *Multibit tree *is an extension of the Singlebit tree, where we no longer demand that all children of a given node are split according to the value of a single bit. In fact we only demand that the data is arranged in *some *binary tree. The match-bits of a given node are computed as all bits that are not a match-bit in any ancestor and for which all fingerprints in the leaves of the node have the same value. Note that a node could easily have no match-bits. When searching through the Multibit tree, the query bitstring *A *is compared to the match-bits of each visited node and *m*_00_, *m*_01_, *m*_10 _and *m*_11 _are updated accordingly.  is computed the same way as  and only branches for which  ≥ *S*_min _are visited.

**Figure 5:**
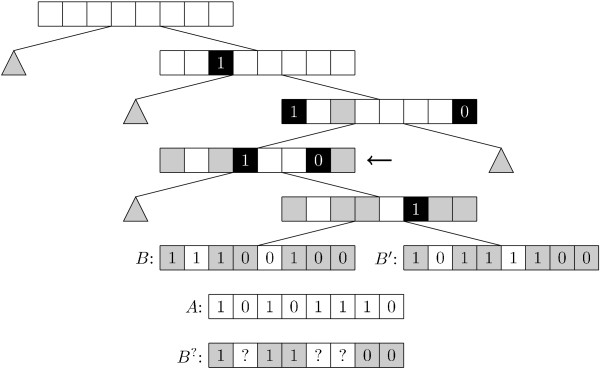
**Multibit tree**. An example of a Multibit tree. The black squares marks the match-bits and their annotation. Grey squares show bits that were match-bits at an ancestor. Grey triangles are subtrees omitted to keep this example simple. When visiting the node marked by the arrow we get *m*_00 _= 1, *m*_01 _= 1, *m*_10 _= 1 and *m*_11 _= 2, thus . Still *S*_*T *_(*A*, *B*) =  and *S*_*T *_(*A, B'*) = .

Again, the best way to build the tree is not obvious. Currently, the same method as for the Singlebit trees is used. For a node with a given set of fingerprints, choose the bit which has a 1-bit in, as close as possible to, half of the fingerprints. Split the fingerprints into two sets, based on the state of the chosen bit in each fingerprint. Continue recursively in the two children of the node. Figure 5 shows an example of a Multibit tree. To reduce the memory consumption of the inner nodes, the splitting is stopped and leaves created, for any node that has less than some limit *l *children. Based on initial experiments, not included in this paper, *l *is chosen as 6, which reduces memory consumption by more than a factor of two and has no significant impact on speed. An obvious alternative way to build the tree would be to base it on some hierarchical clustering method, such as Neighbour Joining [9].

## 3 Experiments

We have implemented the *kD *grid and the Single- and Multibit tree in Java. The implementation along with all test data is available at

http://www.birc.au.dk/~tgk/TanimotoQuery/.

Using these implementations, we have constructed several search methods corresponding to the different combinations of the data structures. We have examined the *k*D grid for *k *= 1, 2, 3 and 4, where the fingerprints in the buckets are stored in a simple list, a Singlebit tree or a Multibit tree. For purposes of comparison, we have implemented a linear search strategy, that simply examines all fingerprints in the database. We have also implemented the strategy of "pruning using the bit-bound approach first, followed by pruning using the difference of the number of 1-bits in the XOR-compressed vectors, followed by pruning using the XOR approach" from [8]. This strategy will hereafter simply be known as *Baldi*. A trick of comparing the XOR-folded bitstrings [8] immediately before computing the true Tanimoto coefficient, is used in all our strategies to improve performance. The length of the XOR summary is set to 128, as suggested in [8]. An experiment, not included in this paper, confirmed that this is indeed the optimal size of the XOR fingerprint. We have chosen to reimplement related methods in order to make an unbiased comparision of the running times independent of programming language differences.

**Figure 6:**
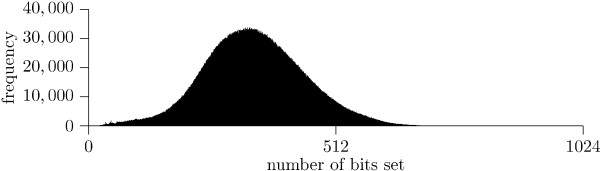
**Distribution of number of bits in fingerprints**. Distribution of the number of bits set in the 1024 bit CDK fingerprints from the ZINC database.

The methods are tested on a real-world data set by downloading version 8 of the ZINC database [1], consisting of roughly 8.5 million commercially available molecules. Note that only 2 million of the molecules have actually been used, due to memory constraints. The distribution of one-bits is presented in Fig. 6, where it can be seen there are many buckets in the 1D grid that will be empty.

The experiments were performed on an Intel Core 2 Duo running at 2.5 GHz and with 2 GB of RAM. Fingerprints were generated using the CDK fingerprint generator [10] which has a standard fingerprint size *N *of 1024. One molecule timed out and did not generate a fingerprint. We have performed our tests on different sizes of the data set, from 100,000 to 2,000,000 fingerprints in 100,000 increments. For each data set size, the entire data structure is created. Next, the first 100 fingerprints in the database are used for queries. We measure the query time and the space consumption.

## 4 Results

**Figure 7:**
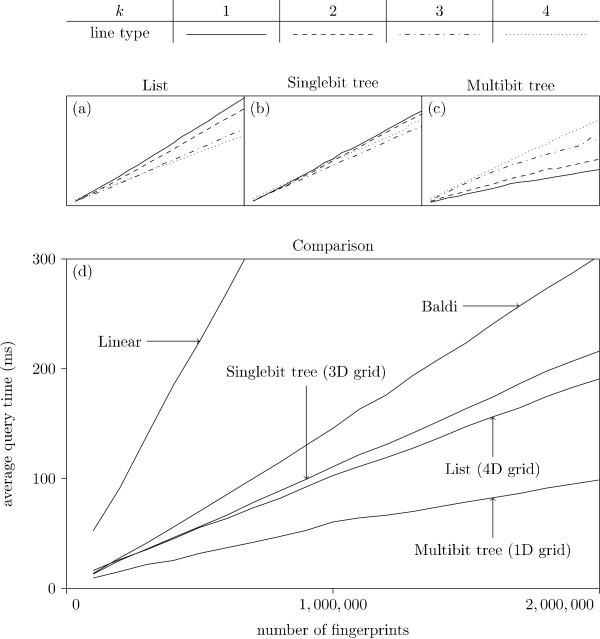
**Average query time, different database size**. Different strategies tested with *k *= 1, ..., 4. Each experiment is performed 100 times, and the average query time is presented. All experiments are performed with a *S*_min _of 0.9. The three graphs (a) - (c) show the performance of the three bucket types for the different values of *k*. The best *k *for each method is presented in graph (d) along with the simple linear search results and Baldi.

Figure 7 shows the average query time for the different strategies and different values of *k *plotted against the database size. We note that the Multibit tree in a 1D grid is best for all sizes. Surprisingly, the simple list, for an appropriately high value of *k*, is faster than the Singlebit tree, yet slower than the Multibit tree. This is probably due to the fact that the Singlebit trees are too small to contain sufficient information for an efficient pruning: the entire tree is traversed, which is slower than traversing the corresponding list implementation. All three approaches (List, Singlebit- and Multibit trees) are clearly superior to the Baldi approach, which in turn is better than a simple linear search (with the XOR folding trick).

**Figure 8:**
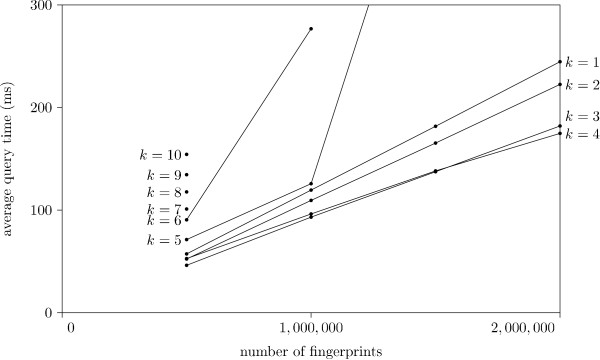
**Average query time on lists, different *k***. Experiments with simple lists for *k *= 1, ..., 10 Each test is performed 100 times, and the average query time is presented. All experiments are performed with a *S*_min _of 0.9. Missing data points are from runswith insufficient memory.

From Fig. 7a we notice that the List strategy seems to become faster for increasing *k*. This trend is further investigated in Fig. 8, which indicate that a *k *of three or four seems optimal. As *k *grows the grid becomes larger and more time consuming to traverse while the lists in the buckets become shorter. For sufficiently large values of *k*, the time spent pruning buckets exceeds the time visiting buckets containing superfluous fingerprints. The Singlebit tree data in Fig. 7b indicates that the optimal value of *k *is three. It seems the trees become too small to contain enough information for an efficient pruning, when *k *reaches four. In Fig. 7c we see the Multibit tree. Again, a too large *k *will actually slow down the data structure. This can be explained with arguments similar to those for the Singlebit tree. Surprisingly, it seems a *k *as low as one is optimal.

**Figure 9:**
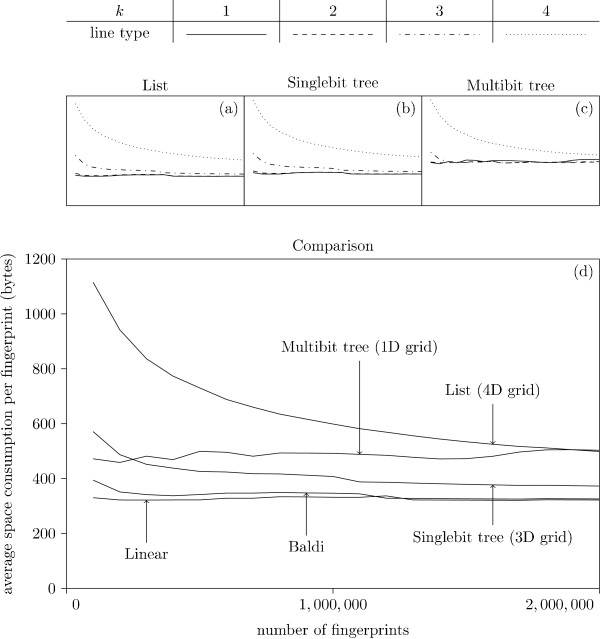
**Average space consumption, different database size**. The memory consumption of the data structure for different strategies tested with *k *= 1, ..., 4. The three graphs (a) - (c) show the performance of the three bucket types for the different values of *k*. The *k *yielding the fastest query time for each method is presented in graph (d) along with the simple linear search results and Baldi.

Figure 9 shows the memory usage per fingerprint as a function of the number of loaded fingerprints. The first thing we note is that the Multibit tree uses significantly more memory than the other strategies. This is due to the need to store a variable number of match-bits in each node. The second thing to note is the space usage for different *k*'s. In the worst case, where all buckets contain fingerprints, the memory consumption per fingerprint, for the grid alone, becomes , where *n *is the number of fingerprints in the database. Thus we are not surprised by our actual results.

**Figure 10:**
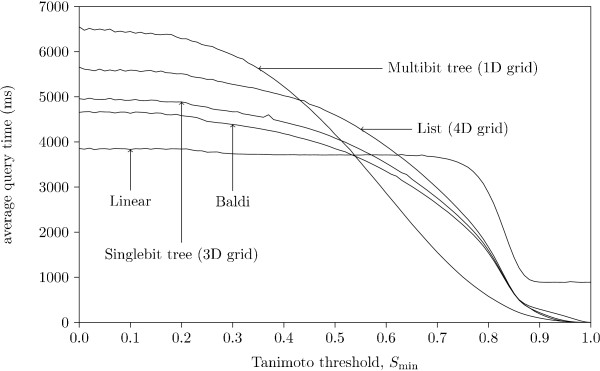
**Average query time, different threshold**. The best strategies from Fig. 7 tested for different values of *S*_min_. All experiments are performed 100 times, with 2,000,000 fingerprints in the database, and the average query time is presented.

Figure 10 shows the search time as a function of the Tanimoto threshold. In general we note that the simpler and more naive data structures performs better for a low Tanimoto threshold. This is due to the fact that, for a low Tanimoto threshold a large part of the entire database will be returned. In these cases very little pruning can be done, and it is faster to run through a simple list than to traverse a tree and compare bits at each node. Of course we should remember that we are interested in performing searches for similar molecules, which means large Tanimoto thresholds.

The reason why linear search is not constant time for a constant data set is that, while it will always visit all fingerprints, the time for visiting a given fingerprint is not constant due to the XOR folding trick.

**Figure 11:**
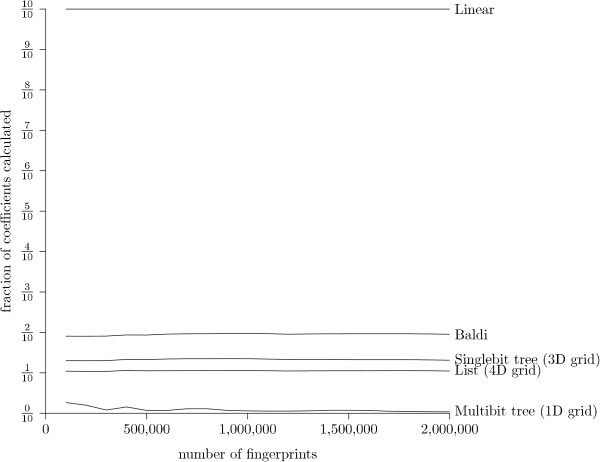
**Fraction of coefficients calculated, different database size**. The fraction of the database for which the Tanimoto coefficient is calculated explicitly, measured for different number of fingerprints. The Tanimoto threshold is kept at 0.9.

The running times of the different methods depend on the number of Tanimoto coefficients between pairs of bitstrings that must be calculated explicitely. This number depends on the method and not on the programming language in which the method is implemented, and is thus an implementation independent performance measure. Figure 11 presents the fraction of coefficient calculated for varying number of fingerprints and a Tanimoto threshold of 0.9. Each method seems to calculate a fairly constant fraction of the fingerprints: only the Multibit tree seems to vary with the number of fingerprints. This is most likely due to the fact that more fingerprints result in larger trees with more information.

**Figure 12:**
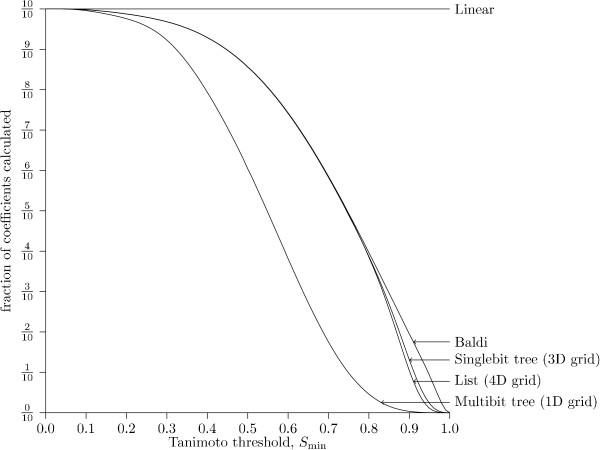
**Fraction of coefficient calculated, different threshold**. The fraction of the database for which the Tanimoto coefficient is calculated explicitly, measured for a varying Tanimoto threshold and 2,000,000 fingerprints.

The result is consistent with the execution time experiments: the methods have the same relative ranking when measuring the fraction of coefficients calculated as when measuring the average query time in Fig. 7. The fraction of coefficients calculated has also been measured for varying Tanimoto thresholds with 2,000,000 fingerprints. The result is presented in Fig. 12. It seems that the relation between the methods is consistent across Tanimoto thresholds. Surprisingly, the Multibit tree seems to reduce the fraction of fingerprints for which the Tanimoto threshold has to be calculated even for small values of the Tanimoto threshold: the three other methods seem to perform very similar up till a threshold of 0.8, whereas the Multibit tree seems to differentiate itself at a threshold as low as 0.2.

The results seems to be consistent with the average query time presented in Fig. 10.

## 5 Conclusion

In this paper we have presented a method for finding all fingerprints in a database with Tanimoto coefficient to a query fingerprint above a user defined threshold. Our method is based on a generalisation of the bounds developed in [6] to multiple dimensions. Our generalisation results in a tighter bound, and experiments indicate that this results in a performance increase. Furthermore, we have examined the possibility of utilising trees as secondary data structures in the buckets. Again, our experiments clearly demonstrate that this leads to a significant performance increase.

Our methods allow researchers to search larger databases faster than previously possible. The use of larger databases should increase the likelihood of finding relevant matches. The faster query times decreases the effort and time needed to do a search. This allow more searches to be done, either for more molecules or with different thresholds *S*_min _on the Tanimoto coefficient. Both of these features increase the usefulness of fingerprint based searches for the researcher in the laboratory.

Our method is currently limited by the rather larger memory consumption of the Multibit tree. Another implementation might remedy this situation somewhat. Otherwise we suggest an I/O efficient implementation where the tree is kept on disk.

To increase the speed of our method further we are aware of two approaches. Firstly, the best way to construct the Multibit trees remain uninvestigated. Secondly, a tighter coupling between the Multibit tree and the *k*D grid would allow us to use grid information in the Multibit tree: in the *k*D grid we have information about each fragment of the fingerprints which is not used in the current tree bounds.

## 6 Competing interests

The authors declare that they have no competing interests.

## 7 Authors' contributions

The project was initiated by TGK, who also came up with the SingleBit tree. JN invented the kD grid and the Multibit tree. All datastructures were implemented, refined and benchmarked by JN and TGK. TGK, JN and CNSP wrote the article. CNSP furthermore functioned in an advisory role.
